# Measurement of cumulative radiation exposure to children and adolescents in contact with outpatients treated with low dose radioactive iodine (^131^I)

**DOI:** 10.1186/s43046-019-0013-0

**Published:** 2020-01-09

**Authors:** Khaled Salman, Shereen Wagieh, Aquib Bakhsh, Tarek Al-Monshy, Omnia Talaat, Musaed Al-Malki, Manal Al-Ezzi, Ashraf Fouda, Gihad Hamid

**Affiliations:** 1 King Abdullah Medical City (KAMC), Makkah, Saudi Arabia; 2 King Abdullah Medical City (KAMC), Jeddah Oncology Centre, Jeddah, Saudi Arabia; 3 National Cancer Institute, Cairo, Egypt; 4 Ministry of Health, Riyadh, Saudi Arabia

**Keywords:** Radiation exposure, Children and adolescents, Outpatients treatment, Low-dose radioactive iodine (^131^I)

## Abstract

**Background:**

Radiation exposure from patients treated with radioactive iodine (^131^I) represents a radiation hazard to children and adolescents, representing the most vulnerable group of household contacts. Our aim was to calculate the cumulative radiation exposure (CRE) figures to children and adolescents sharing the same home with outpatients treated with low-dose ^131^I. The secondary aim was to study the demographic and educational factors that may significantly affect radiation exposure to them.

**Results:**

The whole number of household contacts less than 18 years was 99, out of them 49 ≤ 12 years. CRE level to children and adolescents ranged from 79 to 934 uSv. The mean, median, and 75th percentile figures were 284 ± 178 uSv, 215 uSv, and 334 uSv, respectively. The compliance of this group of contacts to radiation exposure constraint (1 mSv) was 100%. All CRE values were below this figure with 75% of them below half of this constraint. Thirteen adolescents from 12 to 18 years and 17 mothers of 23 household contacts ≤ 12 years got radiation safety instructions (RSI) directly from a radiation safety officer (RSO). This group had a significantly lower mean CRE value (184 ± 93 uSv) compared to those who got RSI from the patient or from other family members (298 ± 185 uSv) with a significant *p* value.

**Conclusion:**

The compliance of adolescents and children to the 1-mSv radiation exposure constraint is 100%. It is advised for adolescents and mothers of children in contact with ^131^I-treated patients to get direct RSI from the RSO, which is the only factor associated with significantly lower radiation exposure figures.

## Background

Radioactive iodine (^131^I) therapy is a widely employed therapeutic modality for patients with Graves’ disease, toxic nodular disease, and well-differentiated thyroid cancer either for postoperative ablation or for treatment of metastatic disease. It has been proven to be a safe and relatively inexpensive therapy. Therapeutic doses of ^131^I commonly range from 100 to 7400 MBq. The lower activity is used for the treatment of toxic goiter, and higher doses are employed to treat metastatic disease in patients with well-differentiated thyroid cancer. The use of higher doses usually requires hospitalization in a special isolation room for few days, and 30 mCi (1110 MBq) was reported as the highest low ^131^I dose approved for outpatient therapy in many countries. It is considered the maximum permissible radioactivity for ambulatory treatment. After ^131^I therapy, the patient becomes a potential radiation hazard to other individuals including household contacts [1, 2].

There is a common agreement of 1 mSv/year dose constraint of radiation exposure from patients treated with ^131^I to children and adolescents between different associations concerned with radiation protection [3, 4]. The smaller constraint for children and young adults is due to the fact that the risk of cancer incidence is the highest at a younger age after exposure to radiation [5, 6]. Ionizing radiation is a well-known carcinogen to which children are particularly more vulnerable due to greater cell division in growing and developing tissues with an expected longer lifetime, increasing the chance of repeated exposure and accumulated damage, with resultant higher cancer risk for children [7].

Awareness and concerns about radiation exposure to children and adolescents increased substantially after major radiation accidents. A lot of epidemiological studies have investigated the link between the Chernobyl accident and cancer and had largely focused on malignant diseases in children, specifically thyroid cancer [8–10]. Recently, Yamashita et al. [11] reported that radiation exposure post-nuclear accidents resulted in an increase in the risk of late-onset thyroid cancer, mainly due to the release of ^131^I in the fallout; this risk was particularly elevated in those exposed during infancy, childhood, and adolescence. There are many ways to maximally reduce exposure of children and adolescents to ionizing radiation without sacrificing diagnostic reliability during CT scanning and hybrid imaging [12–15]. However, radiation hazards have made clinicians skeptical about the treatment usage of ^131^I in children with Graves’ disorders [16, 17] despite some reports confirming the safety of this therapeutic modality in young age [18, 19].

Many studies have been published to measure radiation exposure to household contacts of patients treated with ^131^I. They confirmed proper compliance with radiation exposure constraints. Yet, few studies about this issue have been conducted in the Middle East. To our knowledge, this is the first study in the Middle East concerned with the measurement of radiation exposure to children and adolescents sharing the same home with outpatients treated with low-dose ^131^I.

## Aim

Our study aimed at measuring the external cumulative radiation exposure (CRE) in the post-therapy period to children and adolescents who shared the same home with patients treated with low-dose ^131^I on an outpatient basis in the post-therapy period and their compliance to radiation exposure constraint. The secondary aim was to search for any demographic or educational factor that may affect radiation exposure to this vulnerable group of contacts.

## Methods

The current study was conducted after submission to and approval by the institutional ethics committee and IRB committee. Our study was a prospective study conducted from May 2015 to August 2018 on children and adolescents of well-oriented self-dependent adult patients referred for low-dose (≤ 30 mCi) radioactive iodine therapy on an outpatient basis. The maximum low ^131^I dose that can be given on an outpatient basis in many centers including ours is 1110 MBq (30 mCi), on giving higher activity hospitalization in a special isolation room becomes a must. Patients presented for low-dose ^131^I therapy (< 30 mCi) either for the treatment of toxic goiter or for postoperative ablation in patients with well-differentiated thyroid cancer. The prescribed dose for those with toxic goiter ranged from 370 to 740 MBq (10–20 mCi), while for postoperative ablation in patients with well-differentiated thyroid cancer, the dose was 1110 MBq (30 mCi).

The home requirements needed were the presence of a separate bedroom and bathroom to be used by the patient only for few days post-therapy. Also, no pregnant ladies should be in the household area at least 5 days post-therapy together with the willingness of both the patient and all contacts to precisely follow the given radiation safety instructions. The study included two visits.

### First visit

Radiation safety instructions (RSI) were given in details to the patient by the radiation safety officer (RSO). Also, the aim of the study was explained to the patient including measuring external CRE to children and adolescents in the first 5 days post-^131^I intake using thermoluminescent dosimeters (TLDs). The most important instruction for patients is to restrict contact time with household contacts, especially children and adolescents. The patient should use a separate bathroom and bedroom for a variable period of time, ranging from 1 to 2 weeks, depending on his/her clinical status whether toxic goiter or cancer thyroid and on the given ^131^I dose. If the patient is a nursing woman, she should stop breastfeeding post-therapy for her current lactation episode. Female patients in the childbearing period should have a negative pregnancy test prior to therapy, and they have to avoid pregnancy for 6 months to 1 year after receiving ^131^I therapy. Also, patients should use separate dining tools that should be cleaned separately. Instructions for personal hygiene are also important with urination in the sitting position for men and toilets have to be flushed three times after use with proper hand wash after coming from the bathroom. Everything related to patients from linens, cloth, and towels have to be washed separately for 1 week. The RSO highlighted the importance of keeping enough distance between patients’ aid companion and the patient during returning home, transportation, and during daily contact. The distance should be kept at least one to one and a half meters from the patient for a restricted period of time. The more the distance and the less the time of household contact with the patient, the more the reduction of radiation exposure. This is more important for children and adolescents especially in the first week post-therapy to reduce radiation exposure and to achieve the as low as reasonably achievable (ALARA) principle. Further instructions included taking time off from work, restrict the use of public transportation, and avoiding intercourse for variable periods of time, ranging from 1 to 2 weeks, according to different factors including the type of work, the dose given to the patient, and the underlying disease. By the end of this meeting, the participant and his/her family member/s confirmed their understanding of all given instructions and the ability to transfer them to the rest of the family members. For patients who agreed to participate in the study, a consent form was signed. The purpose of using the TLDs to measure CRE together with how and when to be applied was clearly understood. By the end of this visit, a hard copy of RSI was given to be discussed with family members. The patient was scheduled for a second visit for ^131^I therapy. Also, upon agreement to join the study, the patients were asked to be accompanied by one or more of their family members in the second visit for direct education about RSI, preferably adolescents or mothers of children below 12 years living with the patient in the same house.

### Second visit

The patient presented to the nuclear medicine unit for ^131^I therapy, RSI were explained to the patient and accompanied household contact/s in details with answering all raised questions in a simple way. By the end of this visit, the participant and accompanied contact/s confirmed their understanding of all given instructions and ability to transfer RSI accurately to the rest of household contacts including adolescents and children’s mothers. The purpose of using TLDs together with how and when to be applied was also clearly explained with the assurance of understanding all aspects concerned with TLDs. The participant and contact/s were given another printed copy of the instructions to be strictly followed. TLDs were dispensed in sufficient quantity for children and adolescents living in the same household. By the end of this visit, radioactive iodine therapy was given. Post-^131^I therapy, the patient was sent back home in a separate private transportation other than that of accompanied household contact/s, with special precautions for reducing radiation exposure to the driver. On the fifth day post-^131^I therapy, TLDs were collected. A questionnaire including information about the number of household children and adolescents, who was helping more and spending more time with the patient and their relation to the patient, socioeconomic status, and level of education were also collected. The readings of the collected TLDs were done in the Ministry of Health at Personal Radiation Dosage Program at Radiation Protection Department in Riyadh by using the TLD reader. All patients were assured of the complete confidentiality of all study data and that they will be informed about any detected radiation overexposure.

### Statistical analysis

Statistical analysis was carried out on STATA version 13. Numeric variables were presented as the mean and standard deviation or the median and quartiles. Numeric variables were compared between two groups by the Student *t* test if data were normally distributed and by the Mann-Whitney test if not. Data from more than two groups were compared by ANOVA if normally distributed and by Kruskal-Wallis test if not. Categorical variables were presented as percentages and were compared by chi-square test. For comparisons, a two-sided *p* value was set at 0.05.

## Results

The current study included 39 patients treated with low-dose ^131^I on an outpatient basis. Patients’ criteria and given ^131^I dose are shown in Table 1. Seventy-seven percent of patients were females and 59% of patients received ^131^I for postoperative ablation post-total thyroidectomy for well-differentiated thyroid cancer. No statistically significant difference between education levels of thyrotoxic and cancer thyroid patients was detected. The given ^131^I dose for the latter group was significantly higher compared to the dose given to thyrotoxic patients.

**Table 1:** Characteristics and ^131^I dose of treated patients included in the study

Characteristic	All patients (*n* = 39)	Thyrotoxic patients (*n* = 16)	Thyroid cancer patients (*n* = 23)	*p* value
Age, years				
Mean ± SD	43.4 ± 15.0	38.3 ± 13.7	69.9 ± 15.3	0.081
Range	16.0–85.0	16.0–61.0	28.0–85.0	
Gender, *n* (%)				
Male	9 (23.1)	6 (37.5)	3 (13.0)	0.075
Female	30 (76.9)	10 (62.5)	20 (87.0)	
Patient’s level of education, *n* (%)				
Illiterate	10 (25.6)	2 (12.5)	8 (34.8)	
Primary	5 (12.8)	3 (18.8)	2 (8.7)	
High school	13 (33.3)	7 (43.8)	6 (26.1)	0.267
University	10 (25.6)	3 (18.8)	7 (30.4)	
Post-graduate	1 (2.6)	1 (6.3)	0 (0)	
Given ^131^I dose, MBq (mCi)				< 0.01*
Range	370–1110 (10–30)	370–740 (10–20)	1090–1110 (29.7–30)	
Mean, MBq	795 ± 329 (21.5 ± 8.9)	527 ± 134 (14.2 ± 3.7)	1099 ± 12 (29.7 ± 0.32)	

*Statistically significant

The included 39 patients had 99 contacts sharing the same home in the post-therapy period aged < 18 years, including 49 children up to 12 years of age. The criteria for those contacts are shown in Table 2. Among them, 70% were sons and daughters of patients. Only 13 adolescents (age 12–18 years) (26%) and 17 mothers of 23 children (age < 12 years) (46.9%) received direct RSI from RSO, while the remaining adolescents and mothers of young children got these RSI from the patient or from another family member. The mean CRE for the whole group was 284 ± 178 uSv, with a range from 79 to 934 uSv and 75th percentile of 334 uSv.

**Table 2:** Characteristics and TLD readings of contacts of patients treated with ^131^I

Characteristic	All family members (*n* = 99)	Contacts of thyrotoxic (*n* = 48)	Contacts of thyroid cancer (*n* = 51)	*p* value
Age (years), mean ± SD	11.9 ± 4.7	12.3 ± 4.2	11.4 ± 5.1	0.383
Gender, *n* (%)				
Male	47 (48.0)	19 (39.6)	28 (55.8)	
Female	52 (52.0)	29 (60.4)	23 (44.2)	0.106
Contact relation to the patient, *n* (%)				
Son/daughter	70 (70.7)	29 (60.4)	41 (78.8)	0.097
Sibling	18 (18.2)	12 (25.0)	6 (11.5)	
Others	11 (11.1)	7 (14.6)	4 (7.7)	
Direct education from RSO, *n* (%)				
- Adolescents (age 12–18) (13 adolescents)	13 adolescents (26%)	7 adolescents (14.5)	6 adolescents (11.8)	0.882
- Mothers of children (17 mothers for 23 children ≤ 12 years)	23 children (46.9% of children < 12 years)	10 children (20.4%)	13 children (26.5%)	
CRE (uSv)				
Mean ± SD	284 ± 178	271 ± 175	296 ± 181	
Median	215	208	234	
Q2–Q3	164–334	160–339	166–391	0.437
Range	79–934	79–854	107–934	

Age and gender of patients had no significant effects on CRE figures of the group of children and adolescents. Also, the patient education level had a non-significant effect on CRE to this group of contacts; yet, the lowest CRE were found in the five contacts of patients who got a post-graduate education with a mean value less than the mean CRE of all others (Tables 3 and 4).

**Table 3:** A comparison of CRE readings according to binary characteristics of patients

	Values of CRE (uSv)			*p* value
	Mean ± SD	Median [quartiles]	75th percentile	
Contacts of patients ≤ 40 years (*n* = 49)	308 ± 198	222	391	0.783
Contacts of patients > 40 years (*n* = 50)	260 ± 153	209	292	
Contacts of male patients (*n* = 27)	298 ± 203	231	317	0.831
Contacts of female patients (*n* = 72)	279 ± 167	209	341

**Table 4:** A comparison of CRE readings according to patient’s educational level

Educational level of patients (number of contacts)	Mean ± SD	Median	75th percentile	*p* value
Illiterate contacts (20)	267 ± 197	208	294	0.123
Primary school contacts (11)	249 ± 115	205	298	
High school contacts (42)	290 ± 189	226	354	
University education contacts (21)	329 ± 168	286	387	
Post-graduate education contacts (5)	237 ± 13	189	267	

Age, gender, and relation to the patient of household contacts were statistically insignificant in correlation with their CRE (*p* > 0.05). The sole factor that proved to have a significant correlation with CRE was for adolescents and children of mothers who attended direct RSI sessions from RSO. Those who attended those sessions had significantly lower CRE compared to adolescents and children of mothers who got RSI from the patient or from another family member, with a mean value of 184 ± 93 uSv and 298 ± 185 uSv, respectively (*p* = 0.038) (Table 5).

**Table 5:** A comparison of CRE readings according to patient and contact characters

Characteristic	Categories (*n*)	Values of CRE (uSv)			*p* value
		Mean ± SD	Median	75th percentile	
Contact gender	Male (47)	311 ± 191	225	373	0.225
	Female (52)	259 ± 163	207	296	
Direct education from RSO	No (37 adolescents and 15 mothers of 26 children ≤ 12 years)	298 ± 185	233	391	0.038*
	Yes (13 adolescents and 17 mothers of 23 children ≤ 12 years)	184 ± 93	192	220	
Contacts relation to the patient	Son/daughter (70)	305 ± 79	225	388	0.196
	Sibling (18)	201 ± 103	206	244	
	Others (11)	287 ± 137	190	376	

*Statistically significant

## Discussion

Patients with thyroid disorders treated with ^131^I represent radiation hazard to household contacts including caregivers and family members. Many studies reported that no radiation overexposure was reported if RSI were applied strictly with radiation exposure figures within the radiation exposure constraints [20–22]. Few studies are there dealing with radiation exposure to children and adolescents sharing the same home with the patient in the post-^131^I therapy period [23–26]. In the current study, we were concerned with external radiation exposure to this vulnerable group of children and adolescents. In our study, CRE to all children and adolescents were less than the 1 mSv constraint, and 75% of them had CRE below 50% of this constraint. No statistically significant difference in CRE of children between contacts of patients treated with ^131^I for toxic goiter and those treated for cancer thyroid.

It was reported by Barrington et al. [25] that about 90% of children, contacts to patients treated with ^131^I were within the 1-mSv dose limit. They concluded that hyperthyroid patients can be treated with ^131^I on an outpatient basis, if they were given appropriate radiation protection instructions; yet, they raise the point that a special concern should be given to children aged less than 3 years, as 6/17 of them had exceeded CRE of 1 mSv limit. In our study, we had only 6 children less than 3 years with CRE figures ranged from 0.079 to 0.571 mSv, with an overall 100% compliance for the constraint of 1 mSv.

On the other hand, Mathieu et al. [27] reported a median CRE of children who were household contacts of thyrotoxic patients treated with ^131^I was 0.13 mSv (18 outpatients received 200–600 MBq) with 88% received less than the constraint of 0.5 mSv compared to 100% of thyroid cancer patients’ relatives group (22 outpatients received 3700–7400 MBq). ^131^I retention in the thyroid gland in thyrotoxic patients was accused for this difference, suggesting the need of more extended and stringent restriction periods according to the degree of residual thyroid activity. In our study, only 23 of the contacts exceeded the limit of 0.5 mSv with CRE in the range from 0.5 to 0.934 mSv, which was still lower than the constraint of 1 mSv; out of these contacts, 11 were contacts of hyperthyroid patients. The compliance to 0.5 mSv constraint in our study was 77% and 80.8% for contacts of hyperthyroid patients and thyroid cancer patients, respectively. In the current study, there are comparable values for this compliance together with insignificant difference in CRE figures between contacts of those with toxic goiter and those with well-differentiated thyroid cancer despite the significantly higher doses of ^131^I given in the latter group. This is attributed to more ^131^I retention in the intact thyroid gland in those with toxic goiter compared to little tracer retention by the small postoperative residual thyroid tissue in patients with thyroid cancer. This difference in tracer retention appears to compensate for the significant difference in the dose given and accused for the comparable exposure figures of contacts of both groups.

Few studies reported radiation overexposure to children who are household contacts of ^131^I-treated patients. Molyvda-Athanasopoulou et al. [26] reported an outpatient who got ^131^I therapy (592 MBq) for her hyperthyroid state. They found that this patient had a 12-year-old daughter who received 7.79 mSv during the first 7 days post-therapy period. It was reported to be unexpected for a child in this age, who is able to understand and comply with given radiation safety precautions, to have such high radiation exposure figure. They suggested that in the presence of children in the house, it is better to leave the house for at least a week if possible, but if this cannot be done due to social reasons, giving ^131^I therapy with hospital admission should be considered [26]. Also, although Cappelen et al. [21] reported exposure figures below the 1-mSv constraint, they reported an overexposure to a two-year-old child whose mother did not comply with the given radiation safety precautions. Besides, a recommendation was raised that patients who share the same bedrooms or bathrooms with family members or mothers who are going to be treated with ^131^I and has no one to look after her children in the post-therapy period should be treated on an inpatient basis by a study conducted on Omani patients [24]. All previous studies advised appropriate radiation protection precautions to be given with particular consideration to instructions for children ≤ 12 years. The aforementioned studies support our recommendation of properly giving RSI by RSO to mothers in details. This should be done especially for the treated mothers, with special emphasis and more details about radiation safety precautions regarding their offsprings. Additionally, we have to be sure about their ability to comply with these instructions or at least confirming the presence of somebody else who can care for their child in the few days post-outpatient ^131^I therapy. Otherwise, if this is not feasible, we agree with other reports as regards the recommendation of giving low-dose ^131^I therapy on an inpatient basis to avoid radiation overexposure to children and adolescents.

Patients and contact factors such as age and gender together with patients’ educational level as well as the relation of the contact to the patient had no statistically significant correlation with CRE (*p* values > 0.05). This goes with what was previously reported by Kuo et al. [28] stating that no factor (e.g., age, sex, renal function, and others) had a significant association with radiation exposure to household family members unless they were in close contact with the patient for a long time. Also, our results are in agreement with what was stated by Martin et al. [29] who confirmed the absence of a significant correlation between household contacts radiation exposure and patient education level.

It was found that adolescents and children of mothers who attended direct RSI sessions given by the RSO had significantly lower CRE figures. This emphasizes the value of getting RSI education directly from qualified professionals. The attendance of these education sessions by both patients and contacts is recommended, being associated with a significant reduction in CRE figures and more importantly ensuring the ability to comply with these instructions and to apply them strictly in the proper way. These recommendations are in agreement with other reports emphasizing the value of RSI and their proper application [24, 30].

## Conclusion

Radiation exposure to all children and adolescents who are household contacts of outpatients treated with low-dose ^131^I is below the radiation exposure constraint of 1 mSv with 75% of them having exposure figures below half of this constraint, raising the compliance to given RSI regarding this vulnerable group of household contacts to 100%. We recommend attending direct RSI education sessions given from qualified professionals by both adolescents and mothers of children who share the same home with ^131^I-treated patients, representing the sole factor that has a significant correlation with lower radiation exposure level.

## Data Availability

The data that support the finding of this study are available from the statistical analysis unit in the Research Department in King Abdulla Medical City (KAMC), but restrictions apply to the availability of these data, which were used under license for the current study, and so are not publically available. Data are however available upon reasonable request and with permission of the Research Department in King Abdulla Medical City (KAMC).

## Abbreviations

^131^I: Radioactive iodine 131
ALARA: As low as reasonably achievable
CRE: Cumulative radiation exposure
RSI: Radiation safety instructions
RSO: Radiation safety officer
TLDs: Thermoluminescent dosimeters
